# From victimhood to agency: women’s digital resilience journeys

**DOI:** 10.3389/fsoc.2026.1849427

**Published:** 2026-10-19

**Authors:** Lufuno Magadani, Mampilo Phahlane

**Affiliations:** School of Computing, College of Science, Engineering and Technology, University of South Africa, Pretoria, South Africa

**Keywords:** digital resilience, digital victimization, feminist theory, online abuse, social media, women’s agency

## Abstract

**Introduction:**

Online abuse has become a persistent feature of digital life, particularly for women who occupy visible spaces online. While existing research has substantially expanded the discourse in documenting the harms of such abuse, less attention has been given to how women continue to participate, adapt, and assert themselves within these same environments. This study examines how women move from experiences of digital victimization towards forms of agency.

**Methods:**

Based on feminist theory and perspectives on digital power, the study adopts a qualitative multiple case study approach. It analyses publicly available experiential data relating to three women, Anita Sarkeesian, Ferial Haffajee, and Bonang Matheba, whose encounters with gendered online abuse have been widely visible. Using publicly available data, including social media posts, interviews, and media coverage, the study traces how their responses to online abuse unfold over time.

**Results:**

The findings show that movement from victimhood to agency is neither immediate nor straightforward. Women often experience online harassment that may result in psychological trauma and damage to both personal and professional reputations, which can leave them in a state of hesitation and self-doubt. However, such experiences can also lead to self-reflection and a change of strategy. In this study, women responded by managing visibility, revising narratives, and engaging audiences strategically. Thus, agency in this context is reflected in women’s purposeful decision-making in terms of how they respond, participate, and sustain digital presence.

**Discussion:**

The study contributes to understanding how women sustain participation in digital spaces despite online abuse and provides insights for developing safer and more inclusive digital environments.

## 1 Introduction

Digital spaces have become an integral part of everyday life, creating new opportunities for communication, learning, work, and social interaction globally (Acs, 2022). New opportunities in almost every sector have emerged for social engagement, educational, and professional lives that create a sense of self-belonging, community, and participation. One of the complete changes is the social role of women and their involvement in online environments and in daily life. Through digital spaces, women are able to share ideas, build networks, and navigate through these platforms independently (Arbor, 2022). However, despite these opportunities, digital spaces have also emerged as areas of intense vulnerability (Antonucci et al., 2022; Arbor, 2022).

According to Pande (2024), online challenges have become increasingly prevalent within contemporary society, exposing women and girls to harassment, surveillance, and intimidation. Warner (2025) indicated that in recent years, major social media platforms such as Facebook, X (formerly Twitter), and Instagram appear to have reached a high point, where levels of toxicity have become difficult to ignore. Sheikh and Rogers (2024) also stated that data from United Nations Women and various global studies indicate that 58% of women and girls have experienced some form of technology-facilitated violence. The number is expected to increase due to the advancement and accessibility of online platforms and digital connectivity (Sheikh and Rogers, 2024).

A substantial body of research indicates that women are the main targets in digital spaces because of their gender (Gallegos et al., 2026). These gendered inequalities are the forms of deliberate harmful actions in digital spaces, such as sexual harassment, body shaming, doxing, cyberstalking, and threats of violence (Koch et al., 2025). Alhaboby et al. (2023) stated that the effects of online abuse are not only emotional distress, but also physical safety, psychological well-being and social participation issues. It is evident that digitally hurtful acts share the same criteria as traditional violence: deliberate intent to cause harm, a power imbalance between the perpetrator and victim, and repetition (Ray et al., 2024). Individuals who encounter physical violence are likewise likely to experience digital abuse (Torp Løkkeberg et al., 2024). Much of the existing scholarship has focused on gender and online abuse, as well as the severity and consequences of digital violence (Agunwa, 2024). Scholars have looked at its damaging effects on women’s mental health, self-esteem, academic performance, and societal implications (Agunwa, 2024). While this body of scholarship has been exposing injustice in digital platforms, it has also portrayed women through the viewpoint of vulnerability and victimhood.

This emphasis on victimhood, although it is important, risks producing an incomplete understanding of their experiences in digital spaces. At the same time, while these perspectives outline possible solutions, they can most probably hide the ways in which women learn to adapt, resist, and reclaim their identity and make their voices heard in online spaces. In many areas of their lives, women are not only enduring abuse but also motivated to acquire mechanisms to cope. Existing scholarship thus only partially captures women’s experiences in digital spaces by overlooking how they develop digital resilience and exercise agency in response to online abuse (Yuliarti, 2025).

Women are creating platforms where they are capable of seeking support, building supportive networks, report perpetrators, create alternative online spaces, and transforming personal pain into collective advocacy (Watson, 2024). These responses show women’s agency and resilience by proving they are not passive victims of digital abuse. Even when prioritizing women’s voices, research frequently neglects the practical ways they resist and endure in everyday life. These processes can be better understood through the concept of digital resilience. Digital resilience is more than just survival; it reflects a person’s ability to rebuild confidence and the capacity to recover, adapt to the effects of damage caused, and continue participating in digital platforms in spite of the challenges encountered (Lestari et al., 2025; Sun et al., 2022). Digital resilience indicates emotional strength, social support, and technological competencies (Lestari et al., 2025). Resilience is not just about women’s ability to go through adversity but also about building systems to support them so that they are not the ones to carry the burden of survival (Dupin et al., 2025; Lestari et al., 2025). Dushkova and Ivlieva (2024) indicated that supporting and empowering women requires considerable attention, which needs a collective effort from communities, organizations and policymakers to create a safer environment for all.

According to Gyamera et al. (2024), the concept of agency, which is equally important, emphasizes women’s ability to make decisions for themselves, have their voices heard, and flourish in restrictive or shifting digital environments. Personal agency is demonstrated in diverse ways, including, but not limited to, strategically joining or stepping back from situations, supporting others, and working together toward a common goal (Loehr, 2022). These forms of agency challenge the assumption that women do not have power in online spaces and start to create new opportunities for empowerment (Gyamera et al., 2024).

This study examines how women build strength online by shifting the focus from victimization to personal agency. It goes beyond documenting digital abuse to explore how women interpret their experiences, find support, and turn vulnerability into strength. The study also examines how women become stronger and more confident through their emotional, social, and cultural experiences. Furthermore, it contributes to a deeper understanding of gender, power and technology by bringing together the lived experiences of women who demonstrate resilience and resistance. It also generates knowledge that may guide scholars, practitioners and policymakers in promoting safer and more inclusive digital spaces.

### 1.1 Research aim and questions

The aim of this study is to examine how women who experience online abuse navigate the transition from digital victimization to resilience and agency by analysing the publicly documented experiences of Anita Sarkeesian, Ferial Haffajee, and Bonang Matheba through a qualitative multiple-case study.

#### 1.1.1 Research questions

This study is guided by the following research questions:

1. What forms of online abuse are experienced by Anita Sarkeesian, Ferial Haffajee, and Bonang Matheba in digital spaces?
2. How do these women respond to experiences of online abuse?
3. What forms of digital resilience and agency emerge across the three cases?
4. What similarities and differences can be identified across the three resilience journeys?

The next section presents the literature review, following the establishment of the research problem and the research questions that guide this study. This review discusses current literature on women’s experiences in digital environments and highlights gaps in current knowledge around resilience and agency. It also provides the theoretical basis for the conceptual framework underpinning this study.

## 2 Literature review

The rise of digital platforms has changed how people interact, creating both opportunities and challenges that continue to shape digital experiences (Buonocore et al., 2024). The use of these new platforms has opened up new opportunities for participation but has also strengthened existing gender inequalities and intensified concerns about online abuse (Buonocore et al., 2024; Lythreatis et al., 2022). Building on this context, the next sections review literature on women’s digital experiences.

### 2.1 Gendered dimensions of online abuse

Online abuse occurs in different forms across social structures, creating different experiences and consequences for those who are targeted (Watson, 2024). Existing scholarship shows that women encounter different forms and patterns that target women because of their gender, and these behaviors follow recurring patterns (Gyamera et al., 2024). Budhani et al. (2025) noted that because online accounts are often used for both personal and professional matters, abuse becomes deeply personal and highly disruptive, attacking individuals in both areas of their lives. Watson (2024) also stated that online abuse directed specifically at women takes multiple forms, including sexual harassment, body shaming, cyberstalking, doxing, AI-generated fake intimate images shared without consent, and threats of sexual violence. These forms of abuse usually target women’s bodies, sexuality, morality, and credibility more than men (Gautam and Yadav, 2026; Hall et al., 2023). When exploring gendered abuse, it is important to note that this type of abuse focuses on humiliating, silencing, or intimidating women by violating their identity (Gyamera et al., 2024). The digital space allows abuse to go viral rapidly and, being borderless, makes it exceptionally difficult to hold perpetrators accountable (Freeman et al., 2022).

Gautam and Yadav (2026) have also urged that victims continue to endure long-term sufferings after the initial incident has passed. Studies document psychological effects including anxiety, depression, fear, sleep disturbances, and reduced self-esteem. To cope with online abuse, women sometimes doubt their participation, avoid controversial topics, tighten privacy settings, or withdraw from digital environments altogether (Huber, 2023; Watson, 2024). This phenomenon of self-censorship undermines democratic participation and restricts women’s visibility in public discourse (Zviyita and Mare, 2024). In the workplace, online abuse can hurt reputations, change career paths, and make people feel less likely to want to be leaders in digital or public roles (O’Meara et al., 2024). Therefore, the impact of online abuse is not only focused on individuals but also on social and structural setups, which are affecting women’s access to opportunities and influence (O’Meara et al., 2024). While significant research has established the incidence and consequences of gendered digital violence, less attention has been paid to how women interpret and navigate these environments where power is not equal (Marganski and Melander, 2021).

### 2.2 Women’s digital participation and risk

Digital participation empowers individuals to actively engage in digital platforms (De Alencar et al., 2024). Thus, opportunities for women to access information, build professional networks, mobilize for social causes, and generate income have expanded (De Alencar et al., 2024). However, women’s digital participation occurs within environments that are neither neutral nor risk-free (Sjöholm, 2024). Hence, the same technology that connects women and brings opportunities also makes them more vulnerable (Castaño-Pulgarín et al., 2021). Research indicates that women who are publicly visible, such as activists, academics, journalists, politicians, and content creators, are more exposed and vulnerable to such aggressive behaviors. Digital visibility is the extent to which individuals can be seen and noticed in digital environments (Posetti and Reid, 2024). Ordinary women are also exposed in their daily lives to digital interactions, including social media, messaging platforms, gaming environments, and online classrooms (Gallegos et al., 2026).

Surveillance also intensifies these risks by giving those who observe power over those being observed. Digital platforms (like Facebook, Google, and TikTok) work by gathering information, watching what women do online, and using computer programs to predict their interests to show targeted content and ads (Haddock et al., 2022). Women may experience monitoring by strangers, acquaintances, intimate partners, or even employers (Haddock et al., 2022; Narayani, 2024). This surveillance makes women feel exposed and defenseless as they feel the pressure of being watched at all times (Haddock et al., 2022). Narayani (2024) indicated that participation is not just offering women access but also making them feel secure and respected.

### 2.3 Conceptualizing digital resilience

Digital resilience is a newer concept that has emerged out of existing resilience studies on how to manage the risks and threats that individuals come across (like psychology, education, and technology) (Sun et al., 2022). Regardless of its expanding application, the definition of digital resilience still lacks a common theme (Bridges et al., 2023). The concept of digital resilience emerged from broader resilience research which is mostly orientated toward addressing online safety and digital risks within technology-mediated environments (Sun et al., 2022). The concept of digital resilience was previously established to protect users from cyberattacks and to protect IT infrastructure (Sun et al., 2022). Early studies on digital resilience were about preparing for and responding to online challenges encountered and how to manage them effectively (Denckla et al., 2020). However, many scholars have approached the subject of digital resilience from a one-sided approach, placing an emphasis on personal characteristics such as coping skills, managing emotions, being positive, and self-efficacy (Hill et al., 2025).

In addition, policymakers seem to equate resilience within ICT security standards, largely because such criteria are readily quantifiable and auditable. Due to the development of digital resilience, scholars are becoming critical on the misconception that it is just a personal characteristic used to navigate predictable and unpredictable challenges (Chen and Chen, 2026). According to Grygorenko and Naydonova (2023), the process of resilience is continuously changing because it usually depends on the social environment and the current situation that surrounds individuals. However, recent studies have expanded the understanding of digital resilience, from which it goes beyond the ability to recover from harmful online experiences (Park and Choi, 2026). Researchers are increasingly conceptualizing digital resilience as an ongoing process through which individuals learn from digital adversity, adapt to changing online environments, and have the ability to perform critical tasks during crises despite continued exposure to digital risks (Dupin et al., 2025; Park and Choi, 2026).

From this perspective, resilience is relational, not just something you do on your own (Sabah et al., 2025). This means that it grows out of shared experiences and exchanges within social circles, family life, community networks, and online platforms (Sabah et al., 2025). For example, supportive online networks that guide, teach, and share problems can strengthen resilience, while weak institutional responses and inadequate platform moderation make positive outcomes difficult to sustain (Surya et al., 2024). Thus, resilience requires both personal effort and supportive systems (Sabah et al., 2025).

There is an ongoing debate about what it really means to ask for resilience in our daily lives and policies (Mingarini and Larsson, 2025). Many critics argue that this focus on being resilient is actually a way to quietly shift the burden of dealing with crises onto individuals while systemic issues remain not fully addressed (Krüger, 2023). In this case, resilience is used as an excuse to demand adaptation, turning a need for systemic change into a burden for the individual (Krüger, 2023). This critique is highly applicable in workplaces or societies where women are expected to endure hostile situations rather than demand the respect and accountability they deserve (Krüger, 2023). From this perspective, digital resilience is demonstrated not only through absorbing the major shock of online threats but also through maintaining visibility, seeking appropriate support, and exercising agency within digital spaces (Sabah et al., 2025). This conceptual shift redefines digital resilience from an individual coping strategy into a proactive, transformative process focused on self-discovery through social support and personal growth (Sun et al., 2022). Building on these perspectives, this study conceptualizes digital resilience as a continuous journey in which women utilize their social support and personal strengths to overcome digital threats and assert their presence online. Similarly, the concept is evolving into a continuous learning process in which experiences of online challenges can become a step toward greater digital strength and independence.

### 2.4 Agency, voice, and feminist scholarship

Feminist scholars have questioned the tendency to see women in a single, limited way (Gurrieri and Drenten, 2025). They argue that describing women only as passive victims of unfair systems overlooks the complexity of their daily lives and agency (Gurrieri and Drenten, 2025; Udenigwe et al., 2026). Although many studies highlight the extent of online abuse faced by women, feminist scholars encourage scholarship to move past the idea of victimhood and explore the different ways to resist, adapt, and thrive (Thompson, 2023).

Feminist studies do not understand agency as unlimited freedom. Rather, it refers to the ability to make choices and exercise personal agency and pursue purposeful goals while respecting and operating within the boundaries set by societal expectations (Grau-Sarabia and Harcourt, 2025). Women’s digital agency may appear in subtle ways, and that includes knowing when to speak up and when to log off, building supportive networks, reporting abuse, and using digital platforms for advocacy (Enock et al., 2026).

Voice is another important factor in this discussion. In digital spaces women are offered different options in terms of sharing experiences and community building, allowing them to forge networks of solidarity (Oksiutycz and Lubinga, 2021). Feminist scholars, such as those referencing Masenya, defined “voice” not merely as the act of speaking or vocalization but as the power to be heard, recognized, and validated within social, political, and cultural contexts (Mokoena, 2024). Oksiutycz and Lubinga (2021) stated that when women are facing online harassment, threats, and deep fakes, it acts as a weapon of systemic intimidation designed to make the personal and professional cost of speaking out too high to bear. When it comes to this point, continuing to speak or finding new ways to speak becomes an act of resistance.

Therefore, digital spaces are not the same for everyone. Feminist research is the reminder that when some women are finding power online, many still feel afraid to be themselves or unsafe online due to digital harm and inequality (Enock et al., 2026). In many cases, social and structural inequalities influence whose voice matters and who is silenced based on unfair assumptions about race, class, and sexuality (Tsatsou, 2022). As indicated by Criss et al. (2025), it is important that our identities and backgrounds should not dictate whose voices are amplified and whose are dismissed. Understanding women’s digital resilience, therefore, means paying close attention to these overlapping and interconnected realities.

### 2.5 Research gaps

Marganski and Melander (2021) stated that although studies on online harassment have increased, several areas remain under-researched. Much of the existing literature focuses on revealing the painful experiences and psychological consequences of digital harm (Marganski and Melander, 2021; Sreeraj et al., 2019). While these efforts have been vital in exposing the extent of the problem, they often focus so much on the struggles that victims face and omit chances to offer real solutions. Campbell and Mannell (2016) argue that women’s lived journeys from harmful experiences to forms of agency, negotiation, and self-definition have received little attention. As is frequently mentioned, there is insufficient research into resilience, and it is not usually examined as a process that unfolds over time (Denckla et al., 2020).

Moreover, current frameworks related to digital resilience are either too individualistic or too abstract to be applied uniformly in practice (Awad and Martín-Rojas, 2024). There is a lack of frameworks that explain how women understand resilience and agency and how these constructions are informed by social, cultural, and institutional contexts (Borges, 2025). According to Kong et al. (2022), resilience needs practical applications, not just a theoretical explanation. Understanding these issues will need hearing the voices of women who are bearing the weight of being side-lined and finding the way forward. In addition, feminist scholarship on digital harm has been successful in drawing attention to structural inequalities, but fewer studies have combined this knowledge with systematic engagement with resilience (Richardson and Wilson, 2024). Although feminist analysis has offered a broad mapping of structural inequalities that produce digital harm, more research is needed to connect these findings to how users are constructing resilience and agency (Agunwa, 2024). Vulnerability and agency are not opposites; they work together to influence each other in reality. Therefore, there is still much to learn about how women manage fear and risk while still showing courage and creativity online.

When research focuses mainly on victimhood, it may unintentionally silence the very voices it seeks to empower (Thompson, 2023). Understanding women’s digital resilience journeys is needed by shifting the focus from analysing data to valuing the human experience and agency of women in digital spaces. By filling this gap, individuals will not just focus on the negative aspects of technology but start understanding how they can participate, hold power, and transform digital spaces. These gaps provide the basis for the theoretical and conceptual framework presented in the next section, which brings together feminist theory and digital resilience to examine women’s journey.

## 3 Theoretical and conceptual framework

This study uses feminist theory and recent studies on digital power to explore the topic. By building upon feminist perspectives on power, knowledge, and women’s social experience (Harding, 1991; Hartsock, 1983), the framework conceptualizes online abuse not as an isolated issue but a systemic mechanism of the marginalization and silencing of women. The framework also views agency as finding ways to act, even when that space is challenging (Oleksiyenko, 2023). To provide this perspective, the framework was developed by synthesizing the literature reviewed in Section 2. Feminist theory provided the analytical framework to analyse unequal power structures, challenge the stereotype of women as passive victims, and understand how women actively reclaim digital spaces for empowerment and self-expression (Pucelj, 2025). Processes of women’s responses to and overcoming of interpersonal adversity online were informed by literature on digital resilience. These complementary perspectives were integrated to provide a holistic understanding of women’s digital resilience. Recognizing both the structural conditions that produce online abuse and the relational processes by which women exercise agency was important.

### 3.1 Understanding victimhood and agency

Victimhood, within social theory, is not simply a description of harm; it is how society ranks humanity based on established power dynamics (Bloy et al., 2025). Hence, feminist standpoint theory argues that knowledge stems from privileged views of social reality gained from the perspective of marginalized groups, particularly women (Barkat et al., 2025). Mdletshe and Makhaye (2025) stated that women become “victims” not only because they experience abuse, but because institutions, platforms, and cultural norms present them mainly as vulnerable. For a long time, feminist scholars have warned that while naming and recognizing victimization is a crucial first step toward justice, focusing solely on these factors can unintentionally narrow how women are seen, limiting the way women are understood by focusing only on their trauma (Alyce et al., 2023; Fleckinger et al., 2025). Therefore, if studies continually highlight damage and trauma, they may limit understanding to pain alone.

Agency is often mistaken for having total freedom or fighting against everything without fear, but that is inaccurate. Yet feminist theory offers a more careful interpretation. Agency is relational and emerges within limits, and it can also be quite strategic (Oleksiyenko, 2023). For instance, when a woman takes charge of her digital life by blocking someone toxic, tightening her privacy, leaning on friends for support, or taking a temporary break from digital platforms, she is exercising agency (Enock et al., 2026). These actions may not change inequality, but they protect women’s dignity, safety, and well-being.

Victimhood and agency are not opposites (Kreft and Schulz, 2022). For instance, a woman can experience digital harm and still act with resilience. She can feel fear and still speak. She can retreat in one moment and find the courage to speak in the next. The study aims to understand how these two experiences exist together. Therefore, this study does not use simple opposites to explain experiences. The study’s theoretical grounding sees digital life as a space where vulnerability and strength exist together and influence each other. Within this study, victimhood is understood not only as the experience of harm but also as a social process shaped by unequal power relations. Women who experience online abuse are often positioned through narratives of vulnerability, while the structural conditions that enable harassment may receive less attention. Therefore, this study examines victimhood alongside the social and technological environments that shape women’s experiences online. Using this perspective, the analysis focuses on how gendered forms of abuse attempt to limit women’s participation, credibility, and visibility in digital spaces.

### 3.2 Gender, power, and digital spaces

Digital spaces are often considered neutral platforms that treat all users the same. According to Richterich (2025), feminist and critical technology scholars argue that technologies are never neutral (Alsaleh, 2024). These structures are not neutral; they are made by people’s shared beliefs, the drive for economic survival or gain, and the everyday practices that shape how these technologies are used (Alsaleh, 2024). Online spaces are a reflection of offline hierarchies (Lythreatis et al., 2022). Gender inequality does not go away in digital spaces; sometimes it becomes worse because people can hide behind screens, content is widely visible, and technology can spread harmful messages (Jose and Sivaraman, 2023).

Gender inequalities can operate directly and indirectly in digital spaces (Jose and Sivaraman, 2023). Women’s credibility may be questioned more quickly (Singh et al., 2025). Their appearance may be scrutinized more intensely (Singh et al., 2025). Their authority may be challenged more aggressively (Singh et al., 2025). Platform algorithms can sometimes promote controversial or harmful content, including comments that are sexist or disrespectful toward women (Jose and Sivaraman, 2023). In this sense, the design of digital systems and social injustice work together and influence one another.

Power also shapes whose voices are protected and whose are dismissed (Ferguson, 2023). The systems for reporting abuse and the responses from platforms and institutions decide whether the abuse is taken seriously or ignored (Cover et al., 2026). When institutions or platforms do not give enough protection, women are left to protect themselves (Faith, 2022). This perspective highlights that digital resilience is influenced both by a person’s inner strength and by the larger systems they operate within (Sun et al., 2022).

### 3.3 Development and purpose of the conceptual framework

The conceptual framework presented in Figure 1 was developed based on the integration of feminist theory and other scholarship on digital resilience. The framework conceptualizes women’s digital resilience within the structural inequalities that affect women in digital environments. The first dimension, Experiences of Online Abuse, focuses on the different forms of digital harm experienced by women. This dimension shows the starting point of the framework by recognizing that experiences of harm indicate threats designed to intimidate women out of public or professional spaces. The second dimension, Power Structures, examines how historical patriarchy and offline social inequalities transferred into the digital platforms. This is the reproduction and reinforcement of gendered power relations through digital platforms.

**Figure 1:**
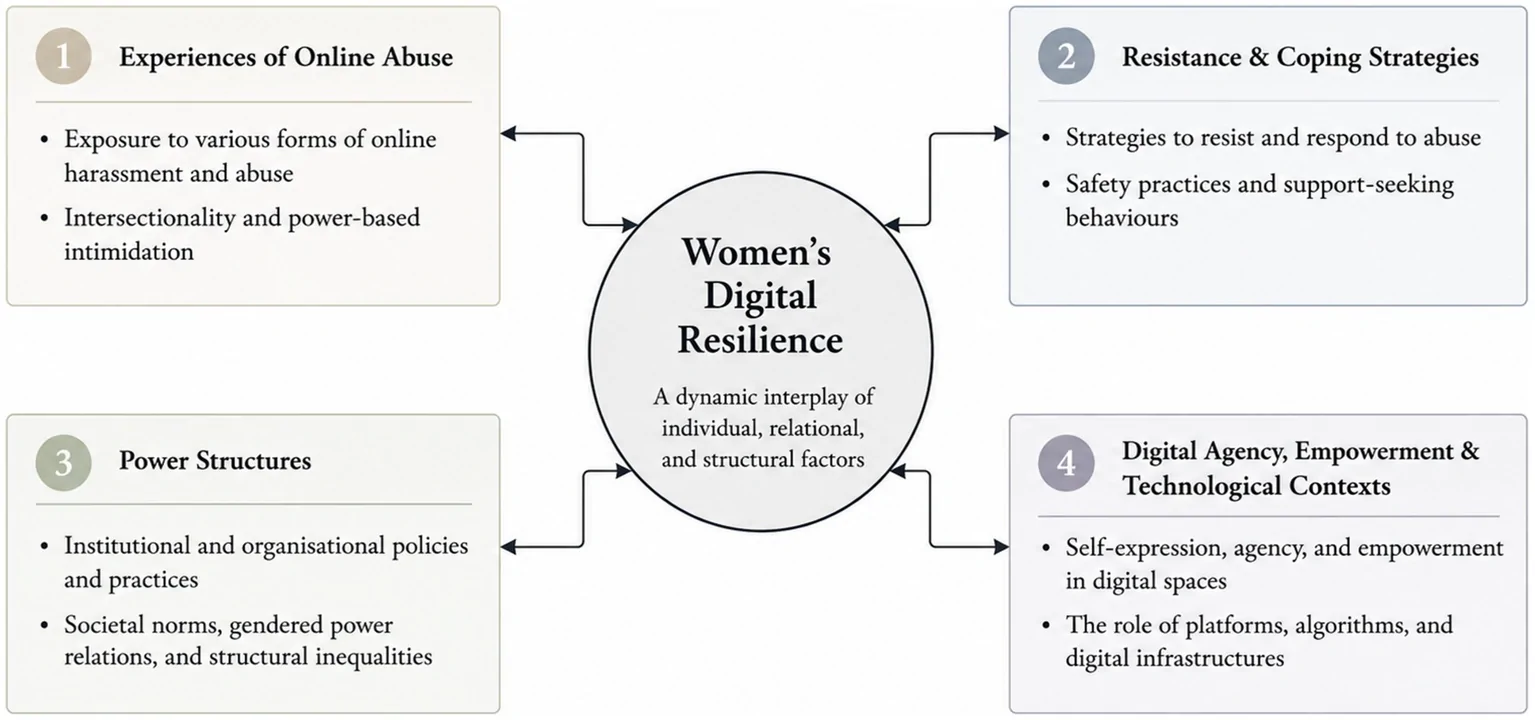
Conceptual framework of women’s digital resilience. Developed by the researchers based on feminist standpoint theory (Harding, 1991; Hartsock, 1983) and digital resilience literature (Sun et al., 2022; Bridges et al., 2023).

The third dimension focuses on resistance and coping strategies. This dimension represents self-preservation as a specific action developed to handle online challenges and maintain well-being. These strategies may include seeking out support from other individuals or temporarily detaching from social networks to protect themselves. The fourth dimension focuses on digital agency, empowerment and technological contexts that define the ability in which women actively define goals and make decisions despite structural barriers. This dimension recognizes that digital agency is not primarily about personal decisions but also opportunities for self-expression.

Together, these four dimensions explain women’s digital resilience as a dynamic and relational process. The framework guided the analysis of the three cases by providing sensitizing concepts for examining experiences of online harm and the conscious decisions to balance their online presence.

In analysing the cases of Anita Sarkeesian, Ferial Haffajee, and Bonang Matheba, this framework examines how online visibility creates both opportunities for participation and exposure to gendered forms of harm. This framework links women’s online abuse to societal power structures, showing how online abuse can be understood not only as a risk to individuals but also as reflecting and reproducing offline structural inequalities. The framework also shows that resilience is not just about personal coping but dealing with systems that are unequal from the start. Building on this conceptual framework, the study used a qualitative multiple-case study design to provide an in-depth exploration of how women negotiate experiences of online abuse while developing resilience and reclaiming agency.

## 4 Methodology

### 4.1 Research design

This study employed a qualitative multiple-case study design to examine how women navigate the transition from digital victimization to resilience and agency following experiences of online abuse. To achieve the main aim of the study, a qualitative approach was selected to examine the subjective meanings, interpretations, and coping strategies reflected in the publicly documented experiences of the participants. Since the research focused on exploring the experiences in depth, a qualitative approach was considered appropriate. Qualitative case study research is particularly suitable for investigating contemporary social phenomena within real-life contexts, especially when the boundaries between the phenomenon and its context are closely intertwined (Yin, 2018).

A multiple-case study design was selected because it involves several case studies to demonstrate replication logic while enabling the researcher to identify recurring patterns, themes, and robust conclusions that strengthen the validity of the research (Stake, 2006; Yin, 2018). The study did not examine a single experience but compared three publicly visible women, Anita Sarkeesian, Ferial Haffajee, and Bonang Matheba, whose experiences represent different professional backgrounds, levels of public visibility, and sociocultural contexts. This comparative approach is intended to provide a rich, holistic understanding of how diverse public personas navigate and respond to intersecting sociocultural pressures.

The study adopted an interpretive research paradigm to allow an overview of the subjective realities, personal narratives, and knowledge creation of these public figures within their unique sociocultural contexts (Creswell, 2014). Cases were selected using purposive, theory-driven sampling. Consequently, the emphasis was not on establishing causal relationships or producing statistically generalizable findings but on developing in-depth narratives of the processes in which women interpret, negotiate, and respond to online harassment.

The qualitative multiple-case design also aligned with the aim of the study. It allowed a systematic examination of the different forms of online abuse experienced by the selected women. This included examining how these women adapted and survived in toxic digital spaces as well as how their resilience journeys align and differ. By examining multiple cases, the study demonstrates the intersection of digital victimization, resilience and agency in different contexts, while recognizing the individual experience of each woman. The study analysed publicly available online content, but it did not use a digital ethnographic approach. Consequently, the research design did not involve participant observation, engagement with online communities and extended immersion in digital environments. As a result, the study adopted a qualitative multiple-case study design based on publicly available documentary sources.

### 4.2 Case selection

Cases were selected using purposive sampling to ensure that the case of each woman could contribute meaningfully to achieving the study’s aim of examining how women transition from digital victimization to resilience and agency. Consistent with the research aim, the selected cases provided rich, publicly documented evidence of online abuse, women’s responses to that abuse, and the development of resilience and agency over time. The study did not select cases for convenience but used purposive sampling to purposefully choose cases that illuminate the phenomenon and allow for analytical comparison across diverse social and professional contexts (Alordiah and Oji, 2024).

Three cases were selected: Anita Sarkeesian, Ferial Haffajee, and Bonang Matheba. These cases represent different levels of global and national visibility. The harassment against Sarkeesian represents a major, internationalized case of digital abuse. Haffajee is a nationally and internationally recognized investigative journalist whose work on politics, corruption, and governance has exposed her to online harassment. Matheba represents the perspective of a celebrity navigating digital reputation and media visibility. The differences in their professional backgrounds and audience size allow the study to examine how digital resilience develops under different levels of public exposure.

Potential cases were first identified by systematically searching trusted news sources, digital media archives, and publicly available online records that documented gendered online abuse. Screening criteria included: (1) documented evidence of coordinated or sustained gendered digital harassment; (2) availability of public statements, posts, or interviews reflecting the woman’s own response; and (3) traceable digital interaction over time enabling process analysis. Cases lacking sufficient public documentation or narrative continuity were excluded.

After the cases were identified, publicly available materials such as social media posts, press statements, interviews, and news reports were collected and arranged in sequential order. Because the study relies exclusively on publicly accessible information, no direct contact with the individuals was required. Following the application of these selection criteria, three information-rich cases were identified. Although each case represents a different professional context and level of public visibility, all three provide well-documented examples of gendered online abuse followed by observable responses that enabled comparative analysis.

Table 1 presents a structured comparison of the three cases. It highlights differences in career background, scale of online abuse, platform use, resilience strategies, and agency outcomes. Comparing different situations helps find common trends while maintaining the unique details of each case.

**Table 1:** Comparative analysis of the selected cases and their documented experiences of online abuse, resilience, and agency.

Cases	Professional context and visibility	Nature of documented online abuse	Scale and platform dynamics	Documented resilience practices	Observable agency outcomes
Anita Sarkeesian	International media critic and feminist commentator operating within the global gaming industry	Coordinated harassment campaign (Gamergate), death threats, doxing attempts, misogynistic hate speech	Cross-platform amplification (YouTube, Twitter/X, forums); migration of abuse across alternative platforms	Continued public speaking; publication of analytical content; advocacy for safer gaming spaces; strategic media engagement	Sustained digital presence; critically analysing how gaming industry structures, hiring, and design practices normalize and reproduce gender inequality; influence on public debate about online misogyny
Ferial Haffajee	Prominent South African journalist, editor, and political commentator with a strong public presence in digital media spaces	online harassment involving gendered abuse, racist attacks, intimidation, and defamatory content linked to her journalism and public commentary	Abuse occurred mainly through social media platforms, particularly Twitter/X,	Legal action against abusive content; public discussion about online harassment; continued journalism and commentary despite attacks; reliance on professional networks and institutional support	Maintained public participation and journalistic voice to conversations about online accountability, digital harassment, and the protection of women in public discourse
Bonang Matheba	Celebrity media personality with strong commercial and brand visibility	Online defamation, viral misinformation, reputational attacks	Platform-wide spread via Twitter/X, blogs, gossip networks	Legal clarification statements; controlled public communication; strategic visibility management	Reputation stabilization; maintenance of commercial partnerships; selective digital engagement

Developed by the researchers based on publicly available sources relating to Anita Sarkeesian, Ferial Haffajee, and Bonang Matheba.

### 4.3 Data corpus and data collection

The study analysed a purposefully selected corpus of publicly available digital materials relating to the three selected cases: Anita Sarkeesian, Ferial Haffajee, and Bonang Matheba. The corpus comprised materials that documented both the incidents of online abuse and the women’s subsequent responses. Multiple public sources were used to develop a contextual understanding of each case. These sources included public posts from X (formerly Twitter), Instagram, and Facebook where available, publicly accessible interviews, press statements, digital news articles, opinion pieces published by reputable media organizations, and other publicly available online records directly related to the documented incidents. The use of multiple data sources enabled the study to capture different perspectives on each case while reducing reliance on a single source of information (Mali, 2023). Searches were conducted using combinations of each participant’s name with terms such as “online harassment,” “online abuse,” “cyberbullying,” “gender-based violence,” “misogyny,” “social media,” “X (formerly Twitter),” and “digital resilience”.

To ensure consistency, only materials that met predefined inclusion criteria were analysed. First, the material had to document a clearly identifiable incident of online hostility, harassment, gendered abuse directed at one of the selected women. Second, it had to provide evidence of the woman’s response, coping strategies, or subsequent actions. Third, the material had to be publicly accessible and originate from the relevant digital platforms or by a credible media organization. Finally, the material had to help explain the development of resilience or agency following the documented online abuse. Materials that merely reported unrelated personal or professional activities, duplicated previously collected information, or lacked sufficient contextual detail were excluded from the analysis.

For each case, materials were collected from the onset of the documented online abuse until evidence of sustained responses and resilience could be identified. The exact period varied across cases depending on when the documented incidents occurred. The study purposefully analysed information-rich materials that directly addressed the research questions and made it possible to make a comparison of all three cases. This approach is consistent in qualitative case study research, where the priority is based on deep analysis over covering every available data point (Alem, 2020; Halkias et al., 2022).

All collected materials were organized chronologically for each case before analysis. This process allowed the researcher to trace the event sequence, analyse the progression of online abuse, track the responses of the women, and compare the three cases. The collected materials were analysed using thematic analysis, focusing on recurring patterns related to online harm, silencing, responses, and resilience. The analysis involved identifying repeated meanings across the three cases and comparing similarities and differences in how each woman experienced and responded to digital abuse. The final corpus consisted of 127 digital materials, distributed across the three cases as follows (Table 2):

**Table 2:** Summary of documentary data sources included in the study.

Data source	Sarkeesian	Haffajee	Matheba	Total documents
X (formerly Twitter) posts	15	10	15	40
Instagram posts	5	5	10	20
Facebook posts	3	0	3	6
Press statements	3	2	3	8
Public interviews	4	3	4	11
News articles	15	12	15	42
Total Corpus	45	32	50	127

Developed by the researchers based on the documentary data collected for this study.

The final corpus of 127 materials was not predetermined before data collection. Materials were collected until sufficient information had been obtained to document the online abuse, responses, and evidence of resilience and agency for each case. Different numbers of materials were included for each case because the volume of publicly available documentation differed considerably across the three women. The study did not seek equal numbers of documents, but prioritized information-rich materials that met the inclusion criteria and addressed the research questions.

### 4.4 Data analysis

To understand the three cases, the publicly available documents were analysed using thematic analysis. Thematic analysis was selected because it provides a flexible and systematic approach for identifying, analysing, and interpreting patterns of meaning within qualitative data (Braun and Clarke, 2019). The initial coding framework was informed by the research questions and conceptual framework, while remaining open to additional themes that emerged from the data during analysis.

Related codes were subsequently grouped into broader themes representing the origins and consequences of online abuse, women’s responses, and expressions of resilience and agency. Using publicly accessible digital sources, the study examined each case step by step: from what first happened to how the woman responded and how her agency and resilience became visible. The analysis therefore combined deductive and inductive coding. Initial coding was informed by the research questions and conceptual framework, while additional themes were allowed to emerge during analysis. The conceptual framework informed the interpretation of the findings by allowing the analysis to remain open to patterns emerging from the data. The analysis began with familiarization with the data. All materials, including social media content, interviews, press statements, and media reports, were reviewed several times to develop an understanding of each case thoroughly. The analysis encompassed message content alongside the strategic dimensions of tone, timing, frequency, and event reframing. Timelines were used to organize the data and trace how the harassment unfolded, document the adaptation of the women’s responses, and identify the manifestation of resilience over time.

Open coding was carried out across all cases. Segments of the text were coded for emerging patterns, including emotional responses, defensive tactics, strategic silence, humor, public reactions, legal actions, and support systems. Deductive coding was informed by the conceptual framework, and inductive coding then permitted new themes to develop from the data that were not anticipated by the framework.

Subsequently, the three cases were compared. At this stage, similarities and differences were carefully examined. Although all three women experienced gendered online hostility, they responded in different ways depending on their level of public visibility, their professional roles, and the support available. This comparison was important because it helped move the study from describing events only to explaining patterns. It led to the development of the process model shown in Figure 1, which brings together the common stages identified across the cases.

To substantiate these findings, the analysis incorporated triangulation across multiple publicly available data sources and cross-references with peer-reviewed publication information relating to each case (Carter et al., 2014). Events, statements, and documented responses were cross-checked across multiple publicly available sources. To ensure getting relevant accuracy, details that could not be corroborated were excluded. Throughout the analysis, the study maintained reflexivity. The interpretations were reviewed and verified many times to guarantee that they were based on facts, not assumptions. The aim was not to praise resilience uncritically, nor to exaggerate harm, but to understand how vulnerability and agency intersect in particular contexts.

### 4.5 Ethical considerations

Although the study uses only publicly available information, throughout the research process the ethical considerations were observed. Reconceptualising public information for academic purposes can still lead to harmful consequences (Bednarek et al., 2024). Online content, even when accessible, exists within specific social and temporal contexts (Setty, 2024). Consequently, the collection and interpretation of publicly available information needed much consideration of its original context. The study relied more exclusively on publicly available materials and did not involve interaction with human participants directly or require the collection of confidential information. All data analysed were obtained from the sources that were publicly accessible, including social media posts, public interviews, press statements, and news reports. The study utilized only publicly available information and did not involve any direct interaction with human participants. As the study relied exclusively on publicly available materials and involved no direct interaction with human participants, no participant recruitment or collection of private information was undertaken. However, ethical principles concerning privacy, contextual integrity, respectful representation and responsible use of publicly available material were respected throughout the study.

Consequently, no primary data were collected from the individuals included in the study. Only publicly available materials from credible and verifiable sources were used. No private accounts, restricted material, or leaked content was included. The selected cases involve publicly recognized individuals whose experiences of online abuse have been widely reported in the public domain. Their identities were not anonymised because the study examined well-documented public cases that could not be meaningfully analysed without identifying the individuals concerned. However, the analysis was limited to information already available in the public domain and did not seek to reveal new personal information.

When discussing abusive material, harmful language was only repeated when absolutely necessary. The intention was to analyse patterns of abuse without increasing offensive or threatening content. Although the materials were publicly accessible, the analysis recognized that they related to sensitive experiences of online harassment and reputational harm. The focus remained on process and structural conditions and not including any personal details that were irrelevant to the research aims. To maintain the aim of the study, irrelevant and sensational descriptions of abuse were omitted; only related information was included. The purpose of the analysis was to understand the progression from online abuse to resilience and agency and avoid reproducing or amplifying harmful material.

The information was cross-checked against multiple publicly available sources for the trustworthiness of the study before it was included in the analysis. Events, statements and documented responses were verified through social media posts, public interviews, press statements and reputable news reports. Information that could not be corroborated by more than one credible source was excluded from the analysis. This approach was to ensure that interpretations were based on verified information and reflected the documented experiences of the three cases in an accurate manner. Ethical considerations were therefore applied throughout both data collection and analysis, ensuring that the study contributes responsibly to discussions on gender, digital harm, resilience, and agency. Following the methodological procedures described above, the findings are presented and discussed in the next section based on the themes generated through the thematic analysis of the documentary data.

## 5 Findings and discussion: journeys from victimhood to agency

The findings are presented according to the main themes identified through the thematic analysis. Table 3 provides an overview of the themes and sub-themes that guided the presentation of the findings.

**Table 3:** Summary of themes and sub-themes identified through the thematic analysis.

Themes	Sub-themes and definitions
Experiences of online harm and silencing	Gendered attacks - Abuse directed at women through gendered insults, stereotypes, or attacks on their identity. Reputational damage - Attempts to harm a woman’s public, professional, or personal reputation through accusations, misinformation, or negative portrayals Coordinated harassment - Repeated or collective online attacks involving multiple users, posts, or platforms. Attempts at silencing - Actions intended to discourage women from speaking or remaining visible in digital spaces.
Emotional and social impacts	Fear - Feelings of insecurity about online or offline harm Emotional distress - Feelings of anxiety, stress, shame, or psychological discomfort resulting from online abuse. Uncertainty - Difficulty in knowing how to respond to abuse. Reputational pressure - Pressure created by concerns about how online abuse may affect professional identity.
Strategic responses and digital visibility management	Selective engagement withdrawal - Choosing when and with whom to respond and not to engage with every abusive interaction. Public communication - Using public statements, interviews, or online communication to respond to issues affecting one’s identity. Boundary setting - Establishing limits on interactions to protect personal and professional space.
Developing digital resilience	Advocacy - Using experiences of online abuse to raise awareness or support change. Professional continuity - Continuing professional work and public participation despite online abuse Support networks - Drawing on colleagues, friends, followers, professional networks, or any other forms of support.
Performing digital agency	Voice - Expressing views and perspectives despite pressures to remain silent. Visibility - Deliberately maintaining or managing one’s presence in digital spaces. Reputation management - Taking deliberate actions to protect or restore one’s public or professional identity. Institutional action - Using legal, organizational, or platform-based mechanisms to respond to online abuse.

Themes and sub-themes were generated from the documentary data analysed in this study.

### 5.1 Experiences of online harm and silencing

Across all three cases, the initial phase of digital abuse was marked by attempts to silence women and undermine their public participation. The harm experienced was not a random insult; it functioned as a form of silencing. Although the contexts are not the same, patterns that are similar have emerged: reputational attack, gendered degradation, and coordinated actions intended to overwhelm the target.

As Anita Sarkeesian’s online activity gained popularity, her platform evolved into a non-profit educational organization dedicated to analysing how modern media intersects with societal issues such as gender, race, and sexuality, encouraging viewers to think critically about media. Reflecting on the nature of the abuse, Sarkeesian described it as “aggressive dogpiles of thousands of voices desperately trying to silence feminist and progressive perspectives” (Sarkeesian, 2019). Sarkeesian explained that online harassment can operate through “conspiracy theories and impersonation” (XOXO Festival, 2014). For example, during the Gamergate controversy, Sarkeesian received repeated threats that affected public appearances and required increased security measures, illustrating how online abuse extended beyond digital platforms into offline professional life (Hern, 2014). In a 2019 post on X, Sarkeesian reflected on the intense backlash she received after expressing concerns about media representations that reinforce patriarchal ideas (Sarkeesian, 2019). She explained that she woke up to thousands of replies, describing them as a “mish-mash of ‘no one cares’ ‘you’re pathetic’ ‘leave our precious toys alone’ ‘relax’ ‘good! women are dumb’ etc etc etc.” (Sarkeesian, 2019). These responses targeted not only her views but also her identity as a woman. What began as an expression of her views evolved into sustained harassment, including threats of violence and organized digital campaigns questioning her credibility. The abuse went beyond personal insults. It disrupted public appearances, prompted security concerns, and attempted to frame her scholarship as illegitimate (Wingfield, 2014). The silencing happened through threats and damage to reputation, sending a wider message that speaking critically in male-dominated online spaces comes at a high cost (Wingfield, 2014). Sarkeesian’s experience illustrates gendered attacks, coordinated harassment, and attempts at silencing as the abuse was directed at her identity and her participation in public digital spaces.

In a similar manner, Ferial Haffajee experienced a different form of online abuse. As an investigative journalist covering politically sensitive issues, she faced coordinated online harassment. This included public questioning of her professional credibility and personal integrity. Many online responses shifted away from her reporting and focused on gendered insults and intimidation aimed at discrediting her work. During periods of her heightened political debate, abusive posts increased across social media, exposing her to sustained public hostility (Friedman, 2019). One documented example involved coordinated online attacks following her reporting on politically sensitive issues related to state capture and corruption. Social media users questioned both her credibility and motives, shifting attention away from the substance of her reporting (Posetti and Reid, 2024). Media interviews and public reporting further indicate that these attacks went beyond criticism of her journalism (Posetti and Reid, 2024; Rodny-Gumede, 2019). For this reason, the attacks became an effort taken to undermine her authority and discourage her from participating in public discourse (Friedman, 2019; Posetti and Reid, 2024; Rodny-Gumede, 2019). Similar to Sarkeesian, the harassment functioned as more than disagreement. It aimed at silencing women whose professional role depended on speaking publicly and holding powerful actors accountable.

In Bonang Matheba’s case, the abuse did not take the same form. The online harm involved false information and attacks on her reputation. One widely publicized allegation claimed that “Bonang is the one that got him into cocaine,” an accusation that later became the subject of a successful defamation lawsuit (Citizen Reporter, 2018). According to media reports, allegations made by Rea Gopane about Matheba went viral across digital platforms and led to a defamation dispute demonstrating how rumors can spread online and put her brand deals and public image at risk (Citizen Reporter, 2018; Gcaba, 2026). The speed at which defamation spread demonstrated how online abuse can increase reputational attacks. For Matheba, the damage to her reputation was more evident because her professional identity and public image were connected to public perceptions of her.

Despite differences between the cases, three common patterns were observed. Online abuse was networked and not limited to a single platform, where harmful content spread across different social media spaces. Tools such as reposts, retweets, shares, and memes helped turn individual attacks into larger, collective forms of harassment. Secondly, gender became another vital factor. Much of the criticism was directed at the three women based on appearance, character, credibility, or emotional behavior. The attacks did not only challenge ideas; they targeted identity. Thirdly, online silencing also worked psychologically and socially. Each case involved difficult moments, like damage to reputation or emotional stress, and high levels of online attention. The harm was therefore not only visible online but also affected professional and personal life. Importantly, this stage of victimhood was not permanent. Although the immediate effects included emotional distress and threats to reputation, these experiences also encouraged a process of adjustment and reflection. The harm women experienced created a need to respond. This suggests that, far from just withdrawing from digital spaces, there was a gradual shift toward more intentional and careful engagement. This initial phase reveals that digital victimhood is produced through repeated patterns aimed at discouraging and intimidating women in public spaces. However, these experiences can also become spaces where resilience begins to develop. Accordingly, Table 4 summarizes the three cases and identifies the main forms of online harm, the initial steps taken by each of the women and the immediate consequences of these experiences. The table focuses on the common and unique features of the cases and then considers the emotional and social consequences in more depth.

**Table 4:** Overview of online harm and initial responses across the three cases.

Case	Online harm	Response	Resilience outcome
Anita Sarkeesian	Gamergate harassment and threats	Advocacy, education, continued participation	Used abuse experiences to advocate for safer gaming spaces
Ferial Haffajee	Online harassment linked to journalism	Continued reporting and public engagement	Strengthened advocacy against online violence
Bonang Matheba	Reputation attacks and misinformation	Legal action and controlled communication	Protected professional identity

Developed by the researchers based on the cross-case thematic analysis.

### 5.2 Emotional and social impacts

Based on the study of three different cases, the first wave of online abuse evoked feelings of shame, embarrassment, and insecurity while also initiating a longer-term process of cumulative trauma and psychological distress. The abuse was not limited to isolated offensive comments; it unfolded as sustained exposure to a toxic and hostile environment that compromised the women’s sense of safety and self-worth. The repeated attacks made the situation worse. What may seem like “just online comments” built up over time and became something much heavier, transforming into a feeling of being constantly watched, judged, and targeted. Sarkeesian explained during interviews that the volume of threats resulted in canceled public appearances and heightened security measures (Hern, 2014). Documented reports from this period confirm that targeted digital harassment forced Sarkeesian and other women in the gaming industry to alter travel, change schedules, and rely on security (Valenti, 2014). Analysis of Haffajee’s public statements following the Equality Court proceedings suggests that prolonged online harassment imposed a significant emotional burden. Similarly, analysis of Matheba’s public responses to online allegations indicates that viral misinformation created sustained reputational pressures (Citizen Reporter, 2018; Rodny-Gumede, 2019). Haffajee described the personal impact of the abuse by explaining, “It has a very chilling impact on you because your phone is an appendage, it’s part of your body now. And so those attacks, they’re personal, they go right into you” (Posetti and Reid, 2024). Her words show that the abuse was not limited to what happened on social media. It also affected her emotionally and became part of her everyday life. Those experiences created a tense and unpredictable environment. Even when no physical harm occurred, the possibility that things could escalate remained. Digital spaces that were once used for work or self-expression began to feel unsafe. Posting, responding, or even logging in required new calculations about risk. The social impacts were equally important. Online harassment hardly remained on one platform. Content was shared across platforms. Screenshots spread quickly. As digital conversations spilled into mainstream media, reputational concerns grew stronger. In highly visible cases, silence could be interpreted as guilt, the same as a response could trigger further attacks. Every action had consequences. These experiences are an indication of issues of fear and emotional distress where online abuse was associated with fear for personal safety and interrupted engagement in everyday digital life.

Across the cases, what appears is not simply a narrative of harm, but both the emotional and social effects functioned as turning points. Also, the cases show uncertainty and reputational pressure. The women had to consider how to react without any further impact on their personal or professional identities. They marked the moment when digital participation could no longer be taken for granted. Once targeted, participation in digital spaces required greater awareness and strategy. The experience prompted reflection and adjustment. Emotional disruption thus became part of the process, not as an ending, but as the starting point for strategic action. The findings suggest that digital victimhood involves more than just harm. It also changes how women see themselves and how they are seen by others. Online visibility becomes difficult to manage. Speaking becomes more cautious. Participation becomes something that must be carefully considered. Understanding these emotional and social effects is important because they create the foundation from which resilience and later expressions of agency develop.

### 5.3 Strategic responses and management of digital visibility

The second theme concerns how the women responded to online abuse. The findings indicate that resilience was expressed through different forms of strategic action without direct confrontation. The women did not respond to every form of harassment but made decisions of knowing when to engage and withdraw and how to maintain their digital presence.

In the case of Anita Sarkeesian, resilience was reflected through continued participation despite sustained harassment linked to the Gamergate controversy. Anita Sarkeesian continued to engage through public communication, advocacy work, and educational content irrespective of challenging experiences she went through (Hern, 2014). Her response was a purposeful choice to stay part of engagement and not be silenced despite premeditated attempts to suppress it. In an interview, Sarkeesian explained that engaging with every harasser was not productive and that protecting her boundaries became necessary when dealing with persistent abuse (Wingfield, 2014; Figure 2).

**Figure 2:**
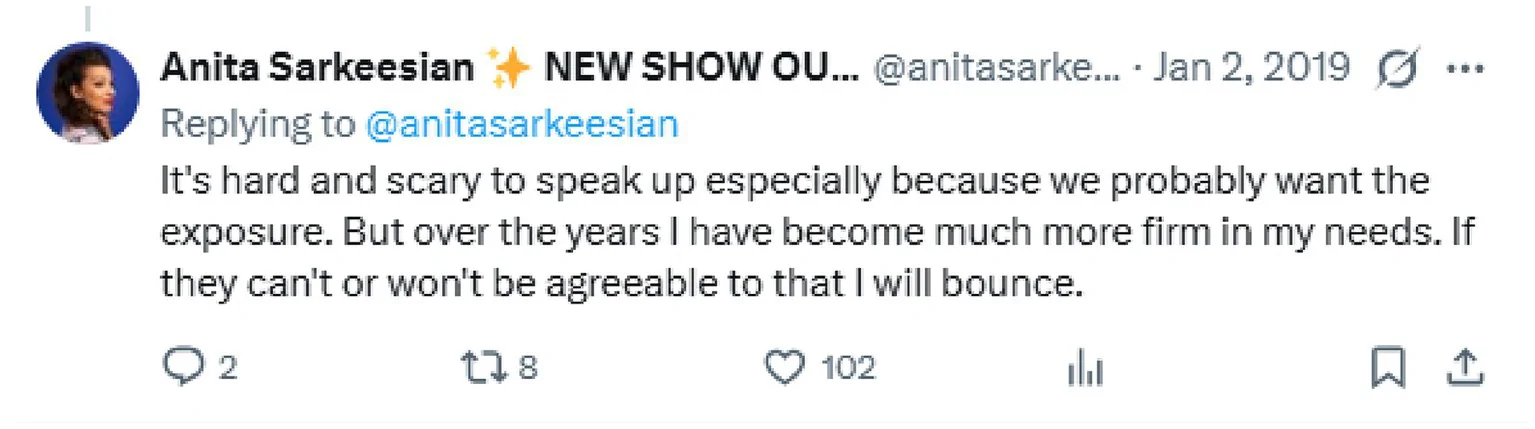
Anita Sarkeesian describing boundary setting and selective engagement withdrawal in response to public engagement. Screenshot of an X post by Anita Sarkeesian, published 2 January 2019. Reproduced with permission.

Sarkeesian’s move to more boldly assert her needs is a sign that resilience did not require a full retreat from public engagement. Instead, she dictated the terms of her participation. This reflects selective engagement and withdrawal, as Sarkeesian chose when to respond and when to disengage from abusive interactions. This suggests that one can make a strategic choice of disengagement as a form of agency, where individuals decide how and where to direct their attention. Accordingly, she never allowed abuse to determine how she participated or engaged online, but she used those platforms to continue discussions around gender and representation.

The responses for Bonang Matheba were an illustration of another form of digital resilience, where communication was manageable and formal clarification was used to address concerns about her reputation (Mbude, 2022). The approach she used reflects the importance of managing visibility within highly public and commercially influenced digital environments.

Ferial Haffajee’s experience demonstrates another form of digital resilience within the context of journalism and public debate. The analysis of her case shows that the choice she made was not centered on withdrawing from digital spaces but on continuing her journalistic work and speaking publicly about online violence against women (Friedman, 2019). Through interviews and public discussions about digital abuse (Rodny-Gumede, 2019), Haffajee gained more courage to transform her personal experience into the debate about online misogyny, press freedom, and the treatment of women in public positions. Her steadfast refusal to be silenced demonstrates that visibility serves as a form of resistance, thereby ensuring female agency within broader advocacy for freedom of opinion and expression (Posetti and Reid, 2024). Her response also illustrates public communication, as she used interviews and public discussion to address online abuse while maintaining her professional role.

All these cases demonstrate that digital resilience involves being strategic about self-regulation and how one participates. For example, Sarkeesian explained that she consciously chose not to continue communicating with some of her most persistent harassers because she would rather not listen to their voices (Hern, 2014). Apart from that, she continued producing content and participating in public discussions despite ongoing harassment (Hern, 2014). This suggests that resilience was an active choice to manage engagement.

### 5.4 Developing digital resilience

The earlier phase was characterized by disruption and attempts to silence women; the next stage across the cases showed adjustment, reflection, and the setting of personal boundaries. Anita Sarkeesian demonstrated her resilience through her continued participation in digital platforms. This change was important because it moved the focus from personal attack to structural issues. Sarkeesian did not opt completely for withdrawal but made these experiences part of a broader public discussion about gender inequality and hostility within gaming communities (Burgess et al., 2017; Wingfield, 2014). In an interview with The Guardian, Sarkeesian described the harassment as “an attack and an assault on women in the gaming industry” and explained that its purpose was to silence and also degrade women who participated in gaming spaces (Hern, 2014). The experiences she encountered highlight that online harassment, particularly during the Gamergate controversy was a deliberate, systemic strategy of misogynistic intimidation aimed at removing women from public gaming spaces (GameQoL, 2012). Her work has transformed these threats into an ongoing discussion on the need for accountability and a more inclusive, safe environment within the digital industry. Resilience involved transforming negative experiences into opportunities for advocacy while also adopting safety measures to balance visibility and protection. Simultaneously, she carefully managed public appearances by increasing security measures to maintain a balance between safety and public visibility. Her advocacy and continuous involvement demonstrate how professional continuity and adaptation can be part of digital resilience.

In the case of Ferial Haffajee, digital resilience developed by becoming persistent and relying on factual reporting. As an investigative journalist who regularly comments on politics and governance, she was regularly targeted after reporting on politically sensitive issues (Posetti and Reid, 2024). One example occurred after Haffajee reported on politically sensitive matters involving state capture and corruption. She received coordinated abuse on X, including accusations that questioned both her credibility and motives (Posetti and Reid, 2024). Haffajee did not engage directly with abusive users but continued publishing investigative work and occasionally corrected misinformation through factual posts and public interviews (Friedman, 2019). In an August 2019 Daily Maverick article, Haffajee described online misogyny as a “rising threat to media freedom.” She further explained that “taking it in and blocking is part of the daily routine now,” illustrating selective engagement withdrawal as part of her practical response to online abuse (Rodny-Gumede, 2019).

For this reason, Haffajee continued publishing her journalism and publicly addressing disinformation only when it threatened the accuracy of public debate, despite responding to every abusive message (Quintal, 2019). In several interviews, she has explained that intimidation and online abuse have become part of the risks associated with investigative journalism but that withdrawing from public engagement would allow harassment to achieve its intended purpose (Quintal, 2019). Her response, therefore, combined selective engagement with continued to maintain professional visibility (Quintal, 2019). Haffajee’s continued journalism also demonstrates professional continuity, while her public discussions of online abuse show how advocacy can emerge from experiences of victimization.

For Bonang Matheba, digital resilience involved strategic reputation management. Responses to public allegations and reputational challenges included official clarification statements, controlled communication, and, where appropriate, legal action (TRUELOVE Digital, 2021; Mbude, 2022). This strategy reflects a different form of resilience. Here, resilience was integrated with brand protection and long-term credibility. Bonang Matheba’s case is an example of institutional action and reputation management. Based on the allegations that linked her to drug use, she responded through legal channels, with her lawyers describing the allegations as defamatory and harmful to her reputation (TRUELOVE Digital, 2021).

Equally important in challenging the reports online, her legal team and management engaged directly with the revenue service to settle the non-compliance issues (Herbst, 2018). Bonang decided to rely on public clarification and her legal team to protect her professional reputation (Citizen Reporter, 2018). The public attention surrounding the allegations and the resulting reputational pressure required a strategic response. The findings show that resilience involved strategic decision-making to avoid immediate reactions. Resilience was context-dependent, shaped by factors such as professional identity, audience size, and access to institutional support. Support from followers, colleagues, or legal teams played an important stabilizing role (Magadla, 2021). Matheba’s use of professional and legal support demonstrates the role of support networks in protecting identity and maintaining professional participation.

In every case, resilience included careful pauses, less posting, and engagement restrictions on digital platforms. The women did not go back to online participation as if nothing had happened; instead, they forged new ways of inhabiting digital space. In this stage, the shift from victimhood begins to materialize. Not because harm disappears, but because digital engagement becomes intentional again. Resilience, as observed across cases, is less about “bouncing back” and more about redefining the terms of participation. Although the women experienced different forms of online abuse, their responses reveal several shared patterns of digital resilience and agency as illustrated in Table 5.

**Table 5:** Cross-case comparison of digital resilience strategies across the three cases.

Case	Main challenge	Resilience strategy	Form of agency
Anita Sarkeesian	Gendered harassment and threats	Advocacy, continued participation, boundary setting	Transforming abuse into public education
Ferial Haffajee	Political harassment and credibility attacks	Continued journalism, selective engagement	Maintaining professional voice
Bonang Matheba	reputational attacks	Reputation management, legal action	Protecting identity and professional reputation

Developed by the researchers based on the cross-case thematic analysis.

The comparison demonstrates that digital resilience is context-dependent. In all three cases, resilience was defined by strategic decision-making that enabled sustained participation in digital spaces in the face of ongoing challenges.

### 5.5 Performing digital agency

The cases demonstrate that agency manifested not only through vocal expression but also through calculated strategic actions within digital environments. For instance, Sarkeesian explicitly chose to document the abuse she received, including rape threats, bomb threats, and death threats. This decision attracted global attention to the severity of online abuse (Burgess et al., 2017; Hern, 2014). This shows voice and visibility because Sarkeesian used her public position to expose online abuse and she did not allow the attacks to stop her participation. Digital agency was not only individual engagement with abuse but also working through official channels and authority structures. This is an instance of Matheba’s case when she chose to seek legal advice, make public statements, give media interviews or report abuse through the platform complaint systems (Magadla, 2021). These actions also demonstrate efforts in reputation management and the need for institutional responses.

Across the cases, digital agency was shown through practical understanding of how digital systems work, using institutional or professional positioning, managing public reputation, and drawing support from social networks. Agency was not articulated in abstract or theatrical manners. The approach was practical, strategic, and strongly established in the material realities of public scrutiny and platform governance. The findings presented above provide a foundation for considering the practical implications of the study and the recommendations that follow.

### 5.6 Cross-case discussion and theoretical contribution

The findings indicate that resilience develops as an evolving process across all three cases, moving through stages of online abuse, reflection, adjustment, strategic participation, and digital resilience and agency. This supports existing research that views resilience as a trend toward a dynamic process conceptualization that moves away from the previous trait-approach. However, the three cases extend this understanding by showing that digital resilience is about how women manage their visibility and make decisions about participation. Sarkeesian decided to continue with her public advocacy while limiting engagement with abusive users. Haffajee kept her professional voice going by using selective engagement and public discussion. Matheba used reputation management, controlled communication and legal steps to protect her image. Such differences show how resilience was not the same in all the women, as it depended on professional position, public visibility, and available support.

The findings also support feminist perspectives that understand agency as a relational approach that addresses structural drivers of inequality to support more sustainable paths toward empowerment and social justice. This study extends this view by showing that agency in digital spaces is not necessarily about direct confrontation. It can mean knowing when to speak, when to withdraw, how to handle visibility, and also when to call for professional assistance. This finding adds a practical dimension to current discussions of women’s digital agency.

The findings further show that resilience and agency are closely connected but are not the same process. Resilience involved adapting to harm and developing ways to remain engaged, while agency became visible through decisions about how that engagement was managed. This view adds to existing work on digital resilience by showing that resilience can create conditions for agency without removing vulnerability. However, even with strategies to remain active on the platform, this implies that women still faced the platform’s limitations and public scrutiny. These strategies support the development of resilience and create opportunities for agency. These findings are brought together in Figure 3, which illustrates how digital resilience and agency develop across the three cases.

**Figure 3:**
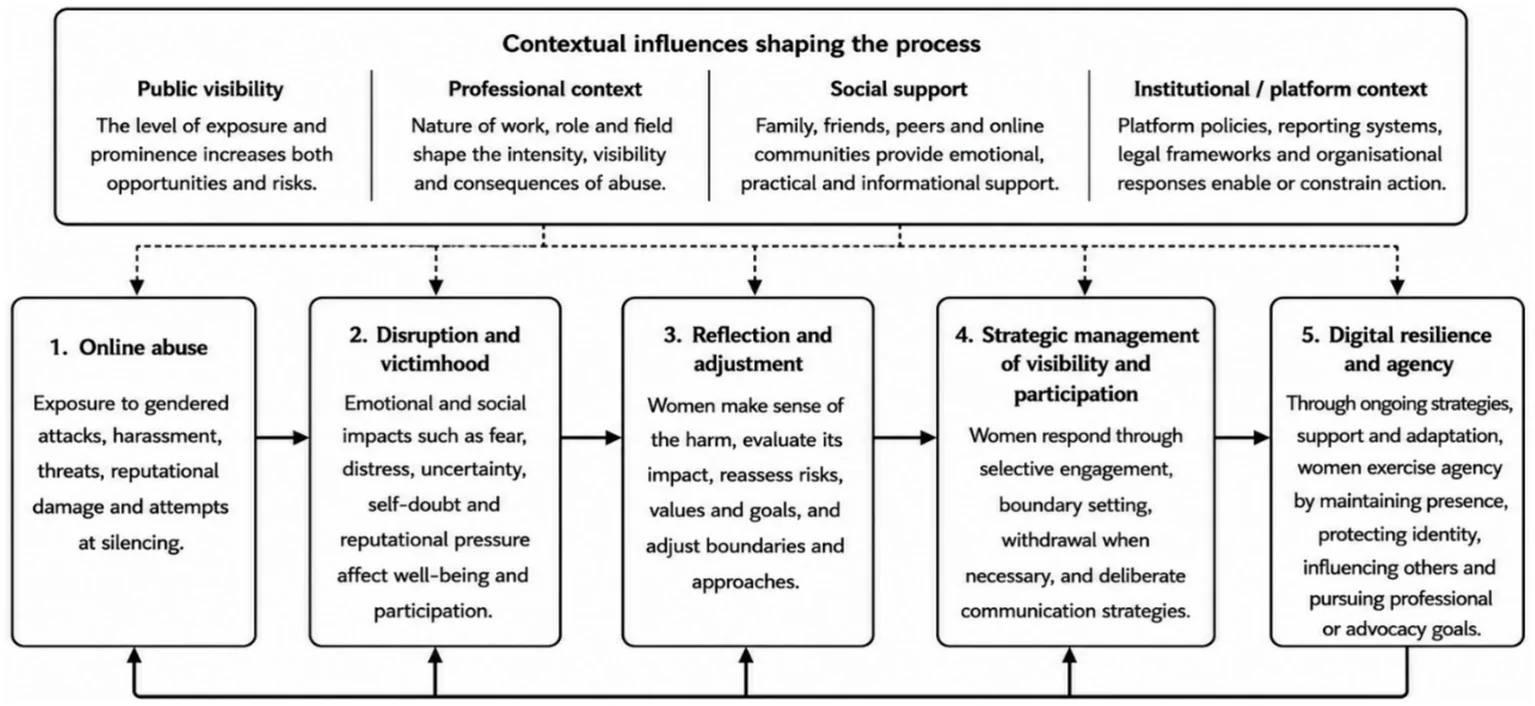
Conceptual framework for understanding digital resilience and agency in response to online abuse. This framework was developed by the authors based on the findings of the present study and informed by previous research on online gender-based violence, agency, empowerment, and digital resilience (Campbell and Mannell, 2016; Sun et al., 2022; Watson, 2024; Hill et al., 2025; Koch et al., 2025; Lestari et al., 2025).

## 6 Implication and recommendations

This study has several important implications. Theoretically, the findings indicate that digital resilience is a persistent adaptation process (Sun et al., 2022). The results also support feminist standpoint theory, suggesting that women’s lived experiences can provide important knowledge about power, agency, and participation in digital spaces (Harding, 1991; Hartsock, 1983). Empirically, the study reveals that the three women from different professional backgrounds who experienced similar patterns of online abuse have developed comparable strategies to remain active in digital spaces. The methodological implications demonstrate that documentary case studies can be used to examine digital victimization through publicly available sources and to identify patterns across different cases. Practically, the findings demonstrated that stronger reporting systems are required, more consistent content moderation, improved digital literacy, and greater institutional support for women experiencing online abuse are also required. These practical implications are discussed below.

One key implication relates to reporting systems. Many victims of online abuse do not use reporting platforms as their first line of response. However, these systems are generally too slow, unclear, or ineffective in addressing gendered harassment (Cover et al., 2026). The study suggests improving the accessibility, transparency, and responsiveness of reporting mechanisms. Platforms should make it easier for users to report abuse, give clear updates after a report is submitted, and review complaints within a reasonable time. Improving these systems would help victims feel that their concerns matter and reduce the feeling of helplessness that often comes with online harassment. Another important implication concerns content moderation policies. Most online platforms have moderation policies, but they are not always followed consistently. Before action is taken, gender-based attacks, harassment, and coordinated trolling often remain visible for extended periods (Watson, 2024; Sjöholm, 2024). Stronger moderation policies are therefore necessary. Platforms should invest in better detection systems and adopt clearer guidelines for identifying and removing abusive content.

The findings also show that digital literacy is important for helping people use online spaces more safely. Many women build resilience by learning how to adjust privacy settings, block abusive users, manage their online reputation, and recognize harmful behavior on the internet. However, these skills are learned through personal experience and do not rely only on formal training. Educational institutions and community organizations should therefore incorporate digital literacy programs that focus not only on technical skills but also on safe and responsible online participation (Yuliarti, 2025; Richardson and Wilson, 2024). In addition, gender awareness and advocacy programs are important for addressing the social attitudes that lead to online harassment. Public campaigns, workshops, and community activities can be impactful in promoting respectful behavior online and supporting people who experience harassment. Further research is needed to understand how race, profession, and public visibility influence women’s experiences of and resilience against online harassment.

These recommendations show that creating safer digital spaces is not just about one or the other; it requires both individual empowerment and structural changes. Empowering women to engage online safely requires improved platform accountability, robust institutional support, and increased public awareness. The study provides valuable insights and practical recommendations, but one must interpret its findings within the context of several limitations. These limitations also point to important directions for future research.

## 7 Limitations and future research

As with all qualitative case studies, the findings of this study are subject to limitations that should be considered. Acknowledging these limitations establishes the study’s boundaries while simultaneously providing a foundation for future research.

The scope of the study is intentionally focused. The study investigates a limited number of publicly recorded cases to explore the processes through which women respond to digital victimization and develop forms of agency. Although this approach allows for detailed and contextual analysis, the findings are not meant to represent the experiences of all women who face online harassment. Digital abuse occurs across multiple platforms and in social and professional settings. Thus, the findings of this study may not be directly applicable to other environments. Therefore, the study emphasizes analytical understanding without broad generalizations about women’s digital experiences.

Another limitation is the sample of cases examined. The selected cases involve women who are highly known public figures. Media reports and digital archives have well-documented their experiences, enabling a systematic analysis. However, women who are less known to the public, including professionals outside the media, students, and casual social media users, may also face different types of online harassment. Differences in social support, institutional protection, or digital literacy may also cause their responses to vary. Future studies should investigate diverse groups of women to have a depth understanding of the development of digital victimization and resilience. The study also faces methodological limitations associated with the use of publicly available data. Media reports and interviews may make online harassment visible, but they fail to reveal the personal and emotional damage (Watson, 2024). Certain personal reactions, coping mechanisms, and offline consequences fall outside the scope of this data analysis. Additionally, digital content is dynamic and may change over time as posts are edited, deleted, or reinterpreted within new contexts (Bednarek et al., 2024). These factors have contributed to how digital experiences are documented and understood.

There are several ways in which future research can build on this study. Researchers may consider combining case study methods with interviews or qualitative fieldwork to understand how women experience and respond to online harassment in their daily lives (Halkias et al., 2022; Alordiah and Oji, 2024). More research is needed to understand how platform design, algorithms, and moderation systems shape the spread and consequences of online abuse. Finally, research must expand to different cultural contexts and social groups to fully map the interaction between gender, technology, and power in digital environments. Prioritizing these areas will advance understanding of women’s online experiences, paving the way for safer and more inclusive digital spaces.

## 8 Conclusion

This study explored how women develop digital resilience and exercise agency in response to experiences of online abuse. The results indicate that online harassment or abuse is not just a solitary digital interaction but also functions as a social phenomenon that undermines reputation, mental well-being, and civic participation. For many women, moments of digital attack create pressure to withdraw or remain silent. However, the cases examined in this study reveal that silence is not the only outcome. Women consistently find ways to navigate digital challenges, reframe their experiences, and stay active online.

The study further indicates that digital resilience is a continuous and evolving process, not only a one-time event or a final recovery stage. To manage harassment, women are setting boundaries by leveraging support networks, adjusting communication, and using digital safety tools. These approaches reflect digital agency that allows women to maintain visibility while also managing online risks. Importantly, this agency does not eliminate vulnerability; rather, it represents a constant balance between being active online and staying safe in public digital space.

At the same time, the findings show that online harassment against women is built into the broader system. While individuals try to protect themselves from online abuse, the responsibility for managing digital violence remains unfairly placed on those who experience it. This situation requires stronger platform governance, policy enforcement systems, improved digital literacy, and stronger legal action against gender-based cyber violence. Effectively addressing online abuse means strengthening individual resilience while restructuring the system of inequalities.

The study contributes to the understanding of digital resilience by demonstrating the relationship between agency and resilience in all the three cases. The results indicate that agency and resilience are closely related but distinct processes. The conceptual understanding of digital resilience extends the discussions by moving past online abuse to the active ways that women engaged within digital spaces.

Finally, this study shows that women’s digital experiences are more than just victim stories. Instead, it illustrates how women actively navigate online spaces, assert their voices, and demonstrate agency in the face of obstacles. Understanding these dynamics is important for creating online spaces where women can participate safely, maintain their dignity, and keep their voices visible and influential.

## Data Availability

The raw data supporting the conclusions of this article will be made available by the authors, without undue reservation.
